# The sustained adverse impact of COVID-19 pandemic on mental health among pregnant women in Sri Lanka: a reassessment during the second wave

**DOI:** 10.1186/s13104-021-05893-1

**Published:** 2022-01-05

**Authors:** Malitha Patabendige, Dhanushka Wanniarachchi, Malika Weerasinghe, Pramith Ruwanpathirana, DMCS Jayasundara, Asanka Jayawardane

**Affiliations:** 1 Base Hospital, Pottuvil, Sri Lanka; 2 Castle Street Hospital for Women, Colombo, Sri Lanka; 3 Consultant Psychiatrist, Colombo, Sri Lanka; 4 National Hospital of Sri Lanka, Colombo, Sri Lanka; 5 Department of Obstetrics and Gynecology, Faculty of Medicine, University of Colombo, Colombo, Sri Lanka

**Keywords:** COVID-19, Pregnancy, Hospitalisation, Anxiety, Epidemiology, Depression, Epidemiology

## Abstract

**Objective:**

To study the change in trend of antenatal mental health and associated factors among a cohort of pregnant women during the second wave of COVID-19 using Hospital Anxiety and Depression Scale (HADS). Previous study using the same scale, during the first wave reported a higher prevalence of anxiety and depression.

**Results:**

A descriptive cross-sectional study was carried out at the two large maternity hospitals in Colombo, Sri Lanka: Castle Street Hospital for Women (CSHW) and De Soysa Hospital for Women (DSHW). Consecutively recruited 311 women were studied. Out of which, 272 (87.5%) were having uncomplicated pregnancies at the time of the survey and 106 (34.1%) were either anxious, depressed, or both. Prevalence of anxiety was 17.0% and depression 27.0%. Overall, continuing COVID-19 pandemic increased antenatal anxiety and depression. The trend was to aggravate depression more intensively compared to anxiety in this cohort of women studied. Special support is needed for pregnant mothers during infectious epidemics taking more attention to antenatal depression.

## Introduction

There is a dearth of studies on continuous surveillance exploring the sustained impact on mental health due to the COVID-19 pandemic in pregnancy. Current evidence suggests that the burden of mental illnesses on pregnancy outcomes is significant [1–3]. The psychological impact among pregnant women in this population during the first wave managed with strict mitigation measures was published earlier [4]. A Sri Lankan study in 2013 has reported a 16.2% prevalence of antenatal depression [5]. Other than that, mental health among the general population in Sri Lanka has not been studied so far. Another study evaluating healthcare providers’ perspectives on maternal mental health showed a lack of application of knowledge into practice [6].

Pregnancy comprises profound physiological changes and a stressor mechanism on the inflammatory system [7]. Due to the weakened immune system, pregnant women are classified as a vulnerable population to contract COVID-19 [8]. In addition, pregnancy might be associated with psychological morbidity including anxiety and depression. These could be related, but not limited to placental hormones, which are considered as stress triggers [9]. With increasing gestational age, levels of these hormones also increase significantly leading to trimester-dependent changes in the mental well-being of pregnant women [10]. Countries with socio-demographic profiles similar to Sri Lanka like Bangladesh and Iran have also reported similar findings recently [11, 12]. Iranian study has shown rising anxiety scores in the third trimester of pregnancy. Another study from Japan has highlighted that concerns about household finances and social support due to lockdown could become targets for interventions among pregnant women to alleviate their mental breakdown [13]. None of the studies has re-evaluated the mental health status of their pregnant women with each wave or changing mitigation measures of COVID-19 which is the main strength of the present study.

The impact of the COVID-19 pandemic on the country’s antenatal care delivery was significant [14]. This study is unique in that, it was conducted during the second wave of increased infections when Sri Lanka responded differently with no lockdown, presenting the trend of psychological impact among a cohort of pregnant women in Colombo, Sri Lanka.

## Main text

### Method

#### Design and setting

A descriptive cross-sectional study was carried out at the two large maternity hospitals in Colombo, Sri Lanka: Castle Street Hospital for Women (CSHW) and De Soysa Hospital for Women (DSHW). Both of these hospitals are the two main public maternity hospitals in the Capital, Colombo, Sri Lanka. The approximate annual delivery rate in CSHW is 11000 livebirths and that of DSHW is 8000. DSHW and CSHW are the first maternity hospitals situated in Sri Lanka and that they are considered model institutions [15, 16].

#### Inclusion/exclusion criteria

All antenatal women attending the antenatal clinics during the second wave of the COVID-19 pandemic (October and November-2020), were invited to participate. Women who were having a pre-diagnosed psychiatric illness, intrauterine fetal demise, history of drug abuse, diagnosed fetal anomaly, and suspected or confirmed COVID-19 infection were excluded.

#### Sample size calculation

The sample size was calculated using sample size formula for cross-sectional studies with a qualitative variable [17]. Type of 1 error of 5% (d), 1.96 as standard normal deviate (Z) and previous prevalence of depression of 19.5% (p) [4] were used yielding a minimum sample size of 242 pregnant women.

#### Methodology

The study instrument was a self-administered questionnaire with two sections. Section 1 assessed demographic and other details potentially affecting their psychological status (loss of income, loss of residence, getting quarantined, close contacts with COVID-19, and pregnancy complications). Section 2 was the locally validated version of the Hospital Anxiety and Depression Scale (HADS) [18]. HADS is a valid and reliable 14-item self-assessment tool to assess the states of anxiety and depression in an outpatient hospital clinic setting [19]. There are seven items for anxiety and depression separately, each item rated on a four-point scale. Scoring for each item ranges from zero to three, with three denoting the highest anxiety or depression level. The scoring method is described in the HADS tool [20]. There are three scoring categories: 0 to 7—Normal; 8 to 10—Borderline and 11 to 21—Abnormal. A total score of ≥ 8 out of 21 (Borderline and Abnormal cases) on the depression or anxiety scale was taken as positive for anxiety or depression [20]. For the current study, a Tamil translation was produced after expert forward and backward translation.

#### Analysis

Descriptive statistics were used to summarise nominal data. Anxiety and depression were calculated according to the responses for the questionnaires. Cross tabulations between the sociodemographic/clinical variables and the anxiety and/or depression were generated. Binary logistic regression was performed to see any significance between psychological disturbance (anxiety and depression) with the demographic and other details as outlined above. P-value < 0.05 was taken as statistically significant. Positive for anxiety and depression were taken as the primary outcome measure in this study.

### Results

Conveniently recruited 311 pregnant women were studied from which 272 (74.3%) were having uncomplicated pregnancies at the time of the survey. Overall, the prevalence of anxiety was 17% (53/311) and depression was 27% (84/311). 106 (34.1%) were either anxious, depressed, or both (entire psychologically disturbed proportion) and 30–39 years age group had the highest psychological disturbance (53/106, 50.0%). Table 1 shows demographic characteristics, the prevalence of anxiety and depression among participants from two hospitals. The differences were not statistically significant between the two hospitals.

**Table 1:** Demographic characteristics, prevalence of anxiety and depression among participants from two hospitals

Variable	DSHW sample, n = 119	CSHW sample, n = 192	Total sample, N = 311	P values
Mean age (SD) in years	28.6 (6.5)	28.9 (5.0)	28.8 (5.7)	0.65^#^
Median parity (IQR)	2 (1–3)	2 (1–2)	2 (1–3)	0.3^¥^
Mean gestational age (SD) in weeks	25.1 (8.9)	23.4 (9.1)	24.3 (9.5)	0.1^#^
Prevalence of anxiety*, n (%)	20 (16.8)	33 (17.2)	53 (17.0)	0.9^¶^
Prevalence of depression*, n (%)	39 (32.8)	45 (23.4)	84 (27.0)	0.07^¶^

^*^as measured by the Hospital Anxiety and Depression Scale (HADS). SD- Standard deviation; IQR- Interquartile range

^#^T test; ^¥^Mann–Whitney *U test; *^*¶*^*Chi-square test*

Details of the binary logistic regression showing association of anxiety and depression with demographic and clinical characteristics are summarised in Table 2. Age, parity, pregnancy complications, loss of income, COVID-19 infection in family members, personal history of quarantine, and loss of residence were not significantly associated with anxiety and depression. The actual numbers were small in each of these subcategories.

**Table 2:** Results of the binary logistic regression showing association of anxiety and depression with demographic and clinical characteristics

Variable (N = 311)	n (%)*	Antenatal anxiety		Antenatal depression		Antenatal anxiety/depression status or both	P value
		aOR (95% CI)	p value	aOR (95% CI)	p value	aOR (95% CI)	
Age in years, mean (SD)	28.8 (5.6)	1.03 (0.96–1.09)	0.47	1.0 (0.95–1.06)	0.98	1.0 (0.95–1.06)	0.73
Parity							
Nulliparous	137 (44.0)	1.32 (0.63–2.80)	0.46	0.87 (0.46–1.63)	0.66	0.81 (0.62–1.07)	0.14
Parous	174 (56.0)						
Complications							
Medically complicated	38 (12.2)	1.3 (0.70–3.8)	0.25	1.5 (0.67–3.24)	0.33	1.88 (0.88–4.03)	0.10
Monthly income in LKR							
< 20,000	108 (34.7)	2.3 (0.39–13.90)	0.35	0.93 (0.19–4.6)	0.92	1.23 (0.30–5.12)	0.78
20,000–49,999	151 (48.6)	1.0 (0.19–5.22)	0.99	1.8 (0.46–6.9)	0.40	1.41 (0.41–4.90)	0.59
> 50,000	19 (6.1)	1.8 (0.38–8.62)	0.46	1.4 (0.36–5.07)	0.65	1.6 (0.48–5.29)	0.45
Missing data	33 (10.6)						
Employment status							
Employed	9 (31.8)	1.1 (0.28–4.56)	0.86	1.92 (0.66–5.57)	0.23	1.24 (0.42–3.71)	0.70
Self employed	37 (11.9)	0.91 (0.41–2.00)	0.81	0.71 (0.36–1.41)	0.33	0.72 (0.38–1.37)	0.32
Unemployed	156 (50.2)	0.97 (0.34–0.27)	0.91	0.84 (0.35–2.03)	0.69	0.8 (0.33–1.90)	0.61
Missing data	19 (6.1)						
Loss of income during the pandemic	51 (16.4)	0.6 (0.26–1.36)	0.22	0.93 (0.53–1.67)	0.80	0.85 (0.50–1.44)	0.54
Loss of residence due to the pandemic	10 (3.2)	1.13 (0.20–6.32)	0.89	2.7 (0.21–3.75)	0.86	0.46 (0.11–1.89)	0.28
Family history of COVID-19 infection	6 (1.9)	0.12 (0.01–1.18)	0.13	–	–	0.55 (0.07–4.23)	0.56
Personal history of recent quarantine	10 (3.2)	9.4 (0.53–165.77)	0.13	0.73 (0.16–3.2)	0.67	4.6 (0.12–177.9)	0.41

^*^age is presented with mean (SD*). aOR* Adjusted Odds Ratio, *CI* Confidence interval, *SD* Standard deviation, *LKR* Sri Lankan Rupees. Antenatal depression due to family history of COVID-19 infection was not computed due to small sample size

### Discussion

This study was conducted to assess the sustained impact of the COVID-19 pandemic [during the second wave at the end of 2020] on the perinatal psychological status among women with no proven COVID-19 infection and to compare the trend of antenatal psychological status with that of during the first wave. It was concluded that psychological disturbance among pregnant women studied was 34.1%. Out of these, 17.0% were anxious and 27.0% were depressed respectively. Our previous study conducted during the first wave [April 2020] showed 17.5% of anxiety and 19.5% of depression [4]. Therefore, the present study clearly shows a rising trend of antenatal depression (19.5% vs 27%) from the first wave to the second wave of the pandemic. Anxiety remained high as in the first wave with no significant change (17.5% vs 17%).

Several previous studies assessing antenatal anxiety and depression among pregnant women without exposure to epidemics have reported similar higher prevalence [21–24]. None of the studies however continued assessment during a pandemic, reporting a trend. Depression and anxiety during pregnancy is major public health concern due to their high prevalence [25]. As noted above a Sri Lankan study has revealed a 16.2% prevalence of antenatal depression as measured by using Edinburgh Postnatal Depression Scale (EPDS) in a cohort of Sri Lankan pregnant women [5]. The present study has revealed a much higher (16.2% *vs* 27.0%) antenatal depression among non-COVID infected pregnant women. A recent meta-analysis evaluating 74 studies has concluded an overall prevalence of anxiety was 42.0% and prevalence of depression was 25% during the COVID-19 pandemic [3]. An Iranian web-based survey in 2021 involving 318 pregnant women has shown that 21% have pregnancy-related anxiety and 42.5% have depression [26]. A study from Qatar has revealed a high prevalence of antenatal anxiety and depression (34.4 and 39.2% respectively) [27]. Studies from South Asia have shown a similar higher prevalence [28–30]. All these studies were cross-sectional studies with no results of reassessments. Therefore, our main strength if the present study is worsening psychological morbidity with continued exposure to the pandemic.

The COVID-19 pandemic has become a global concern and healthcare systems worldwide have been pushed to a breaking point in an attempt to deal with the pandemic. Compared to the previous infectious outbreaks, this pandemic has continued for many months giving rise to a global health and economic burden. This environment can cause sustained anxiety, depression, or psychological trauma [31, 32]. A Japanese study has demonstrated that the impact of household finance and poor social support due to the COVID-19 contributed to worsening psychological well-being [13]. Additionally, pandemic-related issues such as social distancing, isolation, and quarantine, as well as the social and economic fallout can also trigger psychological mediators such as sadness, worry, fear, anger, annoyance, frustration, guilt, helplessness, loneliness, and nervousness [33–35]. For pregnant women, these can add additional psychological burdens. Association of anxiety and depression with demographic and clinical characteristics (Table 2) was noted to be non-significant. The actual sample sizes were small in each category to yield a potential association.

In Sri Lanka, COVID- 19 stringency index was 100 during the first wave with island-wide quarantine curfew and had dropped to nearly 30–50 during the second wave [36]. This study adds to the available literature regarding the significant rising trend of psychological morbidity during successive waves of the COVID-19. Policymakers can take necessary action to rectify these issues.

### Conclusion

This study highlights that the COVID-19 pandemic showed a higher, mixed prevalence of anxiety and depression. The trend was to aggravate depression more intensively compared to anxiety in this cohort of women studied. Special support is needed for pregnant mothers during infectious epidemics taking more attention to antenatal depression.

## Limitations

- Non-probability sampling method.
- Small sample size.
- Hospital-based nature of the study in which prevalence cannot be determined.
- Cross-sectional study design in which cause and effect relationship cannot be built without a control arm.
- Self-reported nature (self-reported tools) of the responses which might be a reason for missing data.
- Inclusion of medically complicated pregnancies.

## Data Availability

Datasets generated from this study will be available from the corresponding author (MP) upon a reasonable request.

## Abbreviations

HADS: Hospital Anxiety and Depression Scale
COVID-19: Corona Virus Disease-2019
CSHW: Castle Street Hospital for Women
DSHW: De Soysa Hospital for Women
EPDS: Edinburgh Postnatal Depression Scale

## References

[1] Tomfohr-madsen LM, Racine N, Giesbrecht GF, Lebel C, Madigan S (2021). Depression and anxiety in pregnancy during COVID-19: a rapid review and meta-analysis. Psychiatry Res.

[2] Alder J, Fink N, Bitzer J, Hösli I, Holzgreve W (2007). Depression and anxiety during pregnancy: A risk factor for obstetric, fetal and neonatal outcome? A critical review of the literature. J Matern Neonatal Med..

[3] Fan S, Guan J, Cao L, Wang M, Zhao H, Chen L (2021). Psychological effects caused by COVID-19 pandemic on pregnant women : a systematic review with meta-analysis. Asian J Psychiatr.

[4] Patabendige M, Gamage MM, Weerasinghe M, Jayawardane A (2020). Psychological impact of the COVID-19 pandemic among pregnant women in Sri Lanka. Int J Gynecol Obstet.

[5] Agampodi SB, Agampodi TC (2013). Antenatal depression in Anuradhapura Sri Lanka and the factor structure of the Sinhalese version of Edinburgh Post Partum Depression Scale among pregnant women. PLoS ONE.

[6] Patabendige M, Athulathmudali SR, Chandrasinghe SK (2020). Mental health problems during pregnancy and the postpartum period : a multicenter knowledge assessment survey among healthcare providers. J Pregnancy.

[7] Challis JR, Lockwood CJ, Myatt L, Norman JE, Strauss JFPF (2009). Inflammation and pregnancy. Reprod Sci.

[8] Rasmussen SA, Smulian JC, Lednicky JA, Wen TSJD (2020). Coronavirus disease 2019 (COVID-19) and pregnancy: what obstetricians need to know. Am J Obs Gynecol.

[9] Ramos IF, Guardino CM, Mansolf M, Glynn LM, Sandman CAHC (2019). Pregnancy anxiety predicts shorter gestation in Latina and non-Latina white women: the role of placental corticotrophin-releasing hormone. Psychoneuroendocrinology.

[10] Coussons-Read M, Okun MSS (2003). The psychoneuroimmunology of pregnancy. J Reprod Infant Psychol.

[11] Saadati N, Afshari P, Boostani H (2021). Health anxiety and related factors among pregnant women during the COVID-19 pandemic: a cross-sectional study from Iran. BMC Psychiatry.

[12] Sharmin N, Lubna Z, Hiramoni FA, Sharker T, Ahmed MZ (2021). Mental health status of pregnant women and breastfeeding mothers during COVID-19 outbreak in Bangladesh. Soc Sci Humanit Open.

[13] Suda T, Miura Y, Katayama M, Senba H, Takahata MNS (2020). Worries and concerns about COVID-19 lockdown aggravate stress reactions among pregnant women. Res Sq.

[14] Patabendige M, Gamage MM, Jayawardane A (2021). The potential impact of COVID-19 pandemic on the antenatal care as perceived by non-COVID-19 pregnant women: women’s experience research brief. J Patient Exp.

[15] Goonewardene M, Dias T (2013). Antenatal care: paradigm changes over the years. Ceylon Med J.

[16] Malitha P, Agampodi SB, Jayawardane A (2021). Perceptions on respectful maternity care in Sri Lanka : study protocol for a mixed-methods study of patients and providers. PLoS ONE.

[17] Charan J, Biswas T (2013). How to calculate sample size for different study designs in medical research?. Indian J Psychol Med.

[18] de Silva D. Anxiety disorders in Sri Lanka. Dissertation submitted to the Postgraduate Institute of Medicine, University of Colombo, Sri Lanka.

[19] Zigmond AS, Snaith RP (1983). The Hospital Anxiety and Depression Scale. Acta Psychiatr Scand.

[20] Rishi P, Rishi E, Maitray A, Agarwal A, Nair S, Gopalakrishnan S (2017). Hospital anxiety and depression scale assessment of 100 patients before and after using low vision care: a prospective study in a tertiary eye-care setting. Indian J Ophthalmol.

[21] Bennett HA, Einarson A, Taddio A, Koren G, Einarson TR (2004). Prevalence of depression during pregnancy: systematic review. Obstet Gynecol.

[22] Ali NS, Azam IS, Ali BS, Tabbusum G, Moin SS (2012). Frequency and associated factors for anxiety and depression in pregnant women: a hospital-based cross-sectional study. Sci World J.

[23] Da Costa D, Larouche J, Dritsa M, Brender W (2000). Sychosocial correlates of prepartum and postpartum depressed mood. J Affect Disord.

[24] Nasreen HE, Kabir ZN, Forsell Y, Edhborg M (2011). Prevalence and associated factors of depressive and anxiety symptoms during pregnancy: a population based study in rural Bangladesh. BMC Women’s Heal..

[25] WHO (2009). Mental health aspects of women’s reproductive health: a global review of the literature.

[26] Hamzehgardeshi Z, Omidvar S, Amoli AA, Firouzbakht M (2021). Pregnancy-related anxiety and its associated factors during COVID-19 pandemic in Iranian pregnant women : a web-based cross-sectional study. BMC Pregnancy Childbirth.

[27] Farrell T, Reagu S, Mohan S, Elmidany R, Qaddoura F, Ahmed EE, Corbett G, Lindow S, Abuyaqoub SM, Alabdulla MA (2020). The impact of the COVID-19 pandemic on the perinatal mental health of women. J Perinat Med.

[28] Shahid A, Javed A, Rehman S, Tariq R, Ikram M, Suhail M (2020). Evaluation of psychological impact, depression, and anxiety among pregnant women during the COVID-19 pandemic in Lahore. Pakistan Int J Gynaecol Obstet.

[29] Hossain MM, Rahman M, Trisha NF, Tasnim S, Nuzhath T, Hasan NT, Clark H, Das A, McKyer EL, Ahmed HU, Ma P (2021). Prevalence of anxiety and depression in South Asia during COVID-19: a systematic review and meta-analysis. Heliyon..

[30] Khatri S, Murthy AK, Hashim U, Kuruthukulangara S, Kumari ALP (2020). Psychological status of pregnant women during COVID-19 pandemic: a cross-sectional study from Mumbai. J Mar Med Soc.

[31] Zandifar A, Badrfam R (2020). Iranian mental health during the COVID-19 epidemic What. Asian J Psychiatr.

[32] Cheung YT, Chau PH, Yip PS (2008). A revisit on older adults’ suicides and Severe Acute Respiratory Syndrome (SARS) epidemic in Hong Kong. Int J Geriatr Psychiatry.

[33] Ahorsu DK, Lin C-Y, Imani V, Saffari M, Griffiths MD, Pakpour A (2020). Fear of COVID-19 scale: development and initial validation. Int J Ment Heal Addict.

[34] Banerjee D (2020). The COVID-19 outbreak: crucial role the psychiatrists can play. Asian J Psychiatr.

[35] Xiang YT, Yang Y, Li W, Zhang L, Zhang Q, Cheung T, Ng CH (2020). Timely mental health care for the 2019 novel coronavirus outbreak is urgently needed. Lancet Psychiatry.

[36] Hale T, Angrist N, Goldszmidt R, Kira B, Petherick A, Phillips T, Webster S, Cameron-Blake E, Hallas L, Majumdar S, Tatlow H.. A global panel database of pandemic policies (Oxford COVID-19 Government Response Tracker). Nat Hum Behav [Internet]. 2021; https://ourworldindata.org/covid-stringency-index 10.1038/s41562-021-01079-8 33686204

